# Cell death inhibitors protect against brain damage caused by cardiac ischemia/reperfusion injury

**DOI:** 10.1038/s41420-021-00698-4

**Published:** 2021-10-23

**Authors:** Suchan Liao, Nattayaporn Apaijai, Ying Luo, Jun Wu, Titikorn Chunchai, Kodchanan Singhanat, Busarin Arunsak, Juthipong Benjanuwattra, Nipon Chattipakorn, Siriporn C. Chattipakorn

**Affiliations:** 1 Neurophysiology Unit, Cardiac Electrophysiology Research and Training Center, Faculty of Medicine, Chiang Mai University, Chiang Mai, 50200 Thailand; 2 Center of Excellence in Cardiac Electrophysiology Research, Chiang Mai University, Chiang Mai, 50200 Thailand; 3 Cardiac Electrophysiology Unit, Department of Physiology, Faculty of Medicine, Chiang Mai University, Chiang Mai, 50200 Thailand; 4 Department of Oral Biology and Diagnostic Sciences, Faculty of Dentistry, Chiang Mai University, Chiang Mai, 50200 Thailand

**Keywords:** Molecular neuroscience, Apoptosis

## Abstract

Cognitive impairment has been reported in patients with myocardial infarction despite a successful reperfusion therapy. Several modes of cell death are involved in brain damage during cardiac ischemia/reperfusion (I/R) injury. Although apoptosis, necroptosis, and ferroptosis inhibitors provided neuroprotection against cerebral I/R injury, the effects of these cell death inhibitors on the brain following cardiac I/R injury have never been investigated. We hypothesized that apoptosis, necroptosis, and ferroptosis inhibitors attenuate brain damage following cardiac I/R injury. One-hundred and twenty-six male rats were used: 6 rats were assigned to sham operation and 120 rats were subjected to 30-min regional cardiac ischemia and 120-min reperfusion. Rats in cardiac I/R group were pretreated with either vehicle (*n* = 12) or one of cell death inhibitors. Rats treated with apoptosis, necroptosis, or ferroptosis inhibitor were subdivided into three different doses including low (L), medium (M), and high (H) doses (*n* = 12/group). Z-VAD, necrostatin-1 (Nec-1), and ferrostatin-1 (Fer-1) were used as apoptosis, necroptosis, and ferroptosis inhibitor, respectively. Rats were sacrificed at the end of reperfusion, and the brain was used to analyze dendritic spine density, Alzheimer’s disease (AD)-related proteins, blood–brain barrier (BBB) tight junction proteins, mitochondrial function, inflammation, and cell death. Our data showed that cardiac I/R led to brain damage and only apoptosis occurred in the hippocampus after cardiac I/R injury. In the cardiac I/R group, treatment with M-Z-VAD and all doses of Nec-1 decreased hippocampal apoptosis and amyloid beta aggregation, thereby reducing dendritic spine loss. M- and H-Fer-1 also reduced dendritic spine loss by suppressing ACSL4, TNF-α, amyloid beta, and tau hyperphosphorylation. Moreover, Bax/Bcl-2 was decreased in all treatment regimen except L-Z-VAD. Additionally, M-Z-VAD and M-Fer-1 partially attenuated mitochondrial dysfunction. Only L-Nec-1 preserved BBB proteins. In conclusion, cell death inhibitors prevented hippocampal dendritic spine loss caused by cardiac I/R injury through different mechanisms.

## Introduction

Acute myocardial infarction (AMI) is a life-threatening cardiovascular disease caused by the blockage of coronary artery [1]. Reperfusion therapy, a standard treatment of AMI, can be achieved by primary percutaneous intervention or fibrinolytic therapy [1]. Unfortunately, adverse sequelae still occurred in the AMI patients despite a successful reperfusion [1]. In addition to cardiac complications, cognitive impairment has been reported in AMI patients [2]. Preclinical data from cardiac ischemia/reperfusion (I/R) injury model revealed that dendritic spine loss also occurred in the brain [3, 4], making it incapable of forming a synapse to maintain normal cognition. In addition, blood–brain barrier (BBB) breakdown is found in the model of cardiac I/R injury [3–5]. The disrupting of BBB allows circulatory inflammation and oxidative stress to get into the brain [6], thus potentiating further brain damage.

Apoptosis, necroptosis, and ferroptosis are associated with cognitive impairment in myocardial infarction [7]. Brain apoptosis was reported in rats with cardiac I/R injury [8, 9]; however, the occurrence of brain necroptosis and ferroptosis has not been investigated. Tumor necrosis factor alpha (TNF-α) can initiate both extrinsic apoptosis and necroptosis [10]. Caspase 8 is a key determinant of the cell fate. If caspase-8 is inhibited, the cell undergoes necroptosis, which is mediated by receptor interacting protein kinases 1, 3 (RIPK1, 3) and mixed lineage kinase domain-like protein (MLKL) [10]. On the other hand, caspase-8 activation and mitochondrial dysfunction induce a cleavage of caspase-3, thereby initiating downstream apoptotic cascade [10]. Iron and lipid peroxide are the main inducers of mitochondrial-independent reactive oxygen species (ROS) production [11], together with an increased long-chain fatty acyl-CoA ligase 4 (ACSL4) and decreased glutathione peroxidase 4 (Gpx4) activity, and they are proposed as mechanisms of ferroptosis [11].

Each cell death inhibitor is developed to inhibit its specific molecular targets. Z-VAD-FMK, an apoptotic inhibitor, is an irreversible pan-caspase inhibitor [12]. Necrostatin-1 (Nec-1) specifically inhibits RIPK1 to reduce necroptosis [13]. A ferroptosis inhibitor, Ferrostatin-1 (Fer-1), scavenges the iron-producing radical products and lipid peroxides [14]. These compounds effectively reduced brain damage in many experimental settings, including traumatic brain injury, cerebral ischemia, ischemic stroke, and subarachnoid hemorrhage [15–18]. However, the effects of these cell death inhibitors on the brain following cardiac I/R injury have not been determined. The hypothesis of this study is that apoptosis, necroptosis, and ferroptosis inhibitors attenuate brain damage in terms of dendritic spine loss, BBB breakdown, Alzheimer’s disease (AD)-related protein expression, mitochondrial dysfunction, inflammation, and cell death following cardiac I/R injury.

## Results

### Cell death inhibitors attenuated hippocampal dendritic spine loss in rats with cardiac I/R injury independently of the cardioprotective effect

**Fig. 1:**
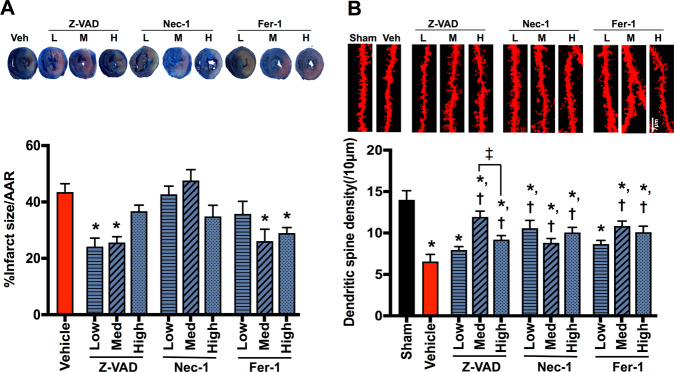
The effects of cell death inhibitors on myocardial infarct size and hippocampal dendritic spine density. **A** Representative images of heart slices using Evans blue-TTC staining (upper panel) and quantitative evaluation of myocardial infarct size/AAR (lower panel, *n* = 6/group); **B** representative images of dendritic spine density (upper panel) and the number of dendritic spines per 10 μm dendrites at hippocampal CA1 region (lower panel, *n* = 8–12/group, 2–3 slides/sample). Data are shown as mean ± SEM. *P < 0.05 vs. Sham, ^†^*P* < 0.05 vs. Cardiac I/R + Vehicle, ^‡^*P* < 0.05 vs. medium dose of Z-VAD. Veh: cardiac I/R rats treated with vehicle; L: cardiac I/R rats treated with low dose of cell death inhibitor; M: cardiac I/R rats treated with medium dose of cell death inhibitor; H: cardiac I/R rats treated with high dose of cell death inhibitor. AAR area at risk, I/R ischemia/reperfusion injury.

Cardiac I/R injury caused myocardial infarction and hippocampal dendritic spine loss, when compared with the sham group (Fig. 1A, B). Treatment with low-to-medium-dose Z-VAD and medium-to-high-dose Fer-1 effectively reduced %infarct size/area at risk, when compared with the vehicle group (Fig. 1A). However, Nec-1, high-dose Z-VAD, and low-dose Fer-1 did not provide this cardioprotective effect (Fig. 1A). Conversely, in the brain, we found that Nec-1 at all doses attenuated dendritic spine loss, and this beneficial effect was also observed in medium-to-high-dose Z-VAD and Fer-1, when compared with the vehicle group (Fig. 1B). Regarding the effects of Z-VAD, medium-dose Z-VAD had higher potency in reducing dendritic spine loss than the high dose (Fig. 1B). These data suggested that cell death inhibitors directly reduced dendritic spine loss independently of cardiac effect. However, low-dose Z-VAD and Fer-1 are insufficient to prevent hippocampal dendritic spine loss following cardiac I/R injury.

### Apoptosis and ferroptosis inhibitors partially attenuated brain mitochondrial dysfunction in rats with cardiac I/R injury

**Fig. 2:**
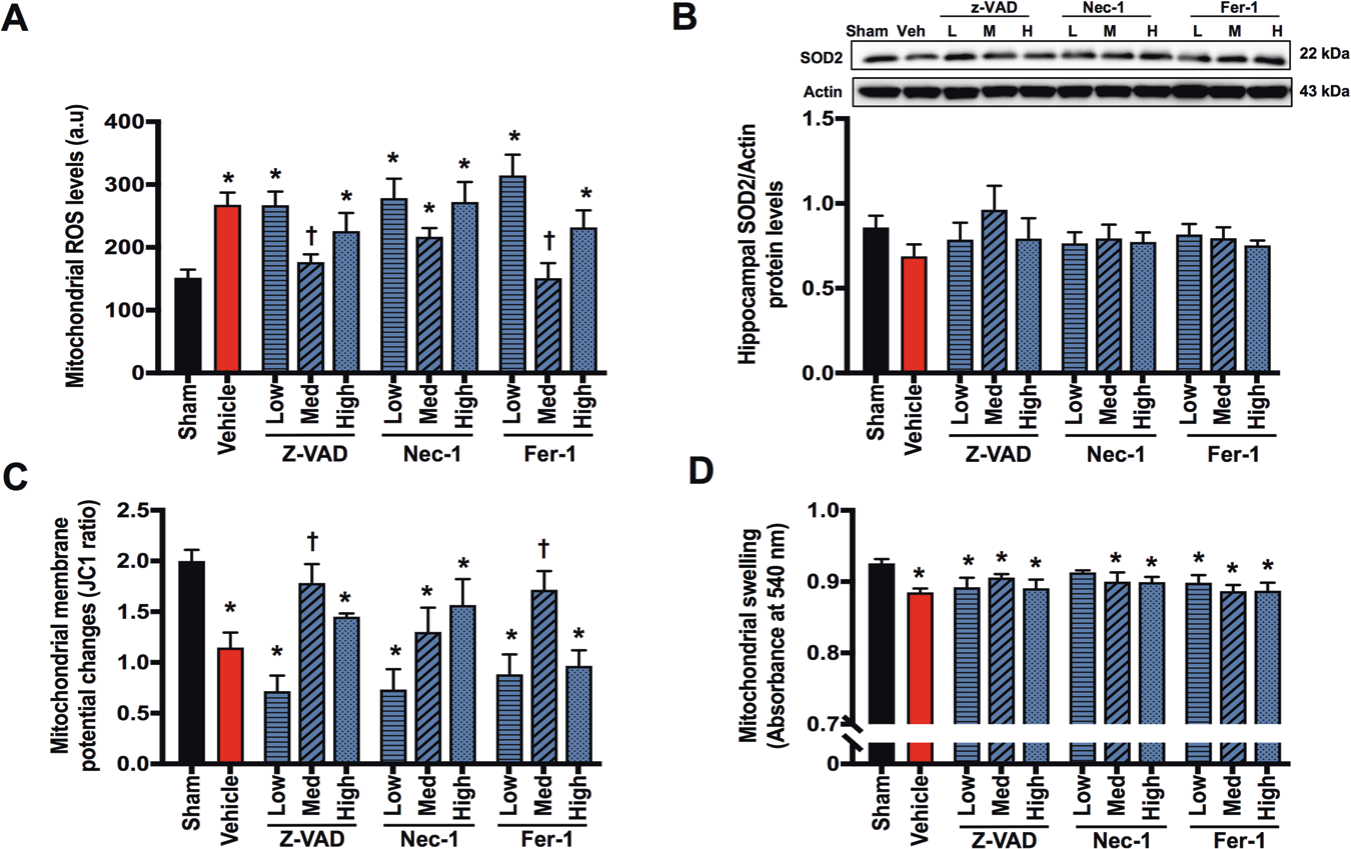
The effects of cell death inhibitors on brain mitochondrial function and brain mitochondrial superoxide dismutase (SOD2) level. **A** Brain mitochondrial oxidative stress (ROS) level (*n* = 8–12/group); **B** brain mitochondrial superoxide dismutase (SOD2) level (*n* = 6/group); **C** brain mitochondrial membrane potential changes (*n* = 8–12/group); **D** brain mitochondrial swelling (*n* = 8–12/group). Data are shown as mean ± SEM. *P < 0.05 vs. Sham, ^†^*P* < 0.05 vs^.^ Cardiac I/R + Vehicle. Veh: cardiac I/R rats treated with vehicle; L: cardiac I/R rats treated with low dose of cell death inhibitor; M: cardiac I/R rats treated with medium dose of cell death inhibitor; H: cardiac I/R rats treated with high dose of cell death inhibitor. I/R ischemia/reperfusion injury.

Brain mitochondrial dysfunction was observed following cardiac I/R injury, as indicated by increased mitochondrial ROS levels, mitochondrial membrane depolarization, and mitochondrial swelling, when compared with the sham group (Fig. 2A–D). However, the levels of mitochondrial superoxide dismutase (SOD2) were not altered (Fig. 2B). Medium-dose Z-VAD and Fer-1 effectively reduced mitochondrial ROS levels and mitochondrial membrane depolarization; however, they did not reduce mitochondrial swelling, when compared with the vehicle group (Fig. 2A, C, D). The other treatment regimen had no effect on brain mitochondrial function (Fig. 2A, C, D). These data suggested that medium-dose Z-VAD and Fer-1 are optimal for attenuating mitochondrial dysfunction, although to a certain extent. None of these inhibitors significantly reduced brain mitochondrial swelling following cardiac I/R injury.

### Cell death inhibitors reduced hippocampal AD-related protein levels, while only Nec-1 prevented BBB breakdown in the hippocampus of rats with cardiac I/R injury

**Fig. 3:**
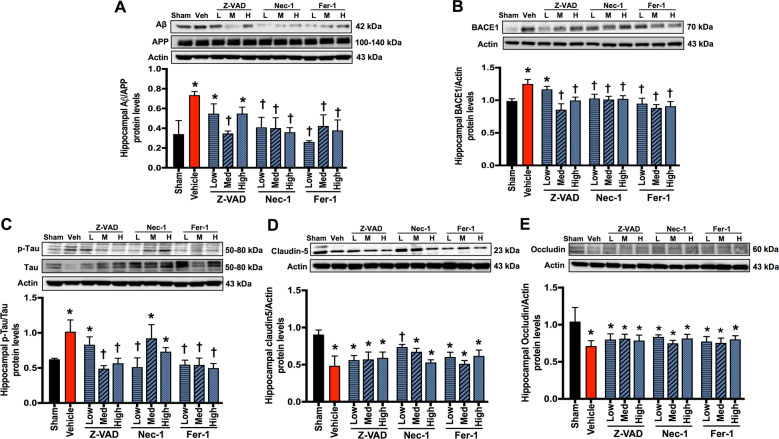
The effects of cell death inhibitors on Alzheimer’s disease-related protein levels and blood–brain barrier breakdown. **A** Hippocampal Aβ/APP protein expression; **B** hippocampal BACE1 protein expression; **C** hippocampal p-Tau/Tau protein expression; **D**, **E** hippocampal tight junction protein claudin 5 (**D**) and occludin (**E**) expression. Data are shown as mean ± SEM. *P < 0.05 vs. Sham, ^†^*P* < 0.05 vs^.^ Cardiac I/R + Vehicle; *n* = 6/group. Veh: cardiac I/R rats treated with vehicle; L: cardiac I/R rats treated with low dose of cell death inhibitor; M: cardiac I/R rats treated with medium dose of cell death inhibitor; H: cardiac I/R rats treated with high dose of cell death inhibitor. I/R ischemia/reperfusion injury, Aβ amyloid beta, APP amyloid precursor protein, BACE β-site amyloid β precursor protein (APP)-cleaving enzyme.

AD-related protein levels were increased following cardiac I/R injury, including Aβ/amyloid precursor protein (APP), β-site amyloid β precursor protein (APP)-cleaving enzyme 1 (BACE1), and p-Tau/Tau, when compared with the sham group (Fig. 3A–C). Medium-dose Z-VAD and all doses of Nec-1 and Fer-1 markedly reduced Aβ/APP protein levels, whereas low- and high-dose Z-VAD did not alter this parameter, when compared with the vehicle group (Fig. 3A). Medium-to-high-dose Z-VAD, along with all doses of Nec-1 and Fer-1, significantly decreased BACE1 protein levels, while low-dose Z-VAD was insufficient to reduce BACE1 protein levels, when compared with the vehicle group (Fig. 3B).

Regarding tau hyperphosphorylation, medium-to-high-dose Z-VAD, low-dose Nec-1, and all doses of Fer-1 suppressed p-Tau/Tau ratio, when compared with the vehicle group (Fig. 3C). Low-dose Nec-1 markedly reduced tau hyperphosphorylation, whereas medium-to-high-dose Nec-1 was ineffective. Collectively, medium-dose Z-VAD, low-dose Nec-1, and all doses of Fer-1 effectively reduced AD-related protein levels and tau hyperphosphorylation in the brain following cardiac I/R injury.

Cardiac I/R injury also caused BBB breakdown as indicated by reduced hippocampal occludin and claudin 5 protein levels, when compared with the sham group (Fig. 3D, E). Our results showed that only low-dose Nec-1 increased claudin 5 protein levels, whereas the other treatment regimen did not affect claudin 5 protein levels, when compared with the vehicle group (Fig. 3D). None of these inhibitors affected occludin protein levels, when compared with the vehicle group (Fig. 3E). These data suggested that only low-dose Nec-1 prevented BBB breakdown following cardiac I/R injury.

### Apoptosis and ferroptosis inhibitors suppressed hippocampal TNF-α protein levels in rats with cardiac I/R injury

**Fig. 4:**
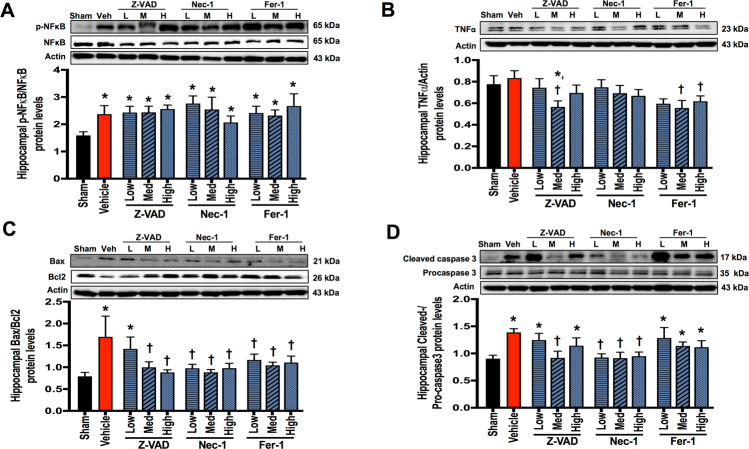
The effects of cell death inhibitors on hippocampal inflammation and apoptosis signaling pathways. **A** p-NF-κB/NF-κB protein expression; **B** TNF-α protein expression; **C** the ratio of Bax/Bcl-2 protein expression; **D** cleaved caspase-3/procaspase-3 protein expression ratio. Data are shown as mean ± SEM. *P < 0.05 vs. Sham, ^†^*P* < 0.05 vs. Cardiac I/R + Vehicle; *n* = 6/group. Veh: cardiac I/R rats treated with vehicle; L: cardiac I/R rats treated with low dose of cell death inhibitor; M: cardiac I/R rats treated with medium dose of cell death inhibitor; H: cardiac I/R rats treated with high dose of cell death inhibitor. I/R ischemia/reperfusion injury.

Cardiac I/R injury caused brain inflammation as indicated by an increased p-nuclear factor-κB (NF-κB)/NF-κB ratio, while TNF-α protein levels were not affected by cardiac I/R injury, when compared with the sham group (Fig. 4A, B). None of these inhibitors affected p-NF-κB/NF-κB protein levels (Fig. 4A). However, medium-dose Z-VAD and medium-to-high-dose Fer-1 suppressed TNF-α protein levels (Fig. 4B), suggesting that apoptosis and ferroptosis interfered brain TNF-α signaling.

### Apoptosis occurred in the hippocampus, which was reduced by apoptosis and necroptosis inhibitors in rats with cardiac I/R injury

**Fig. 5:**
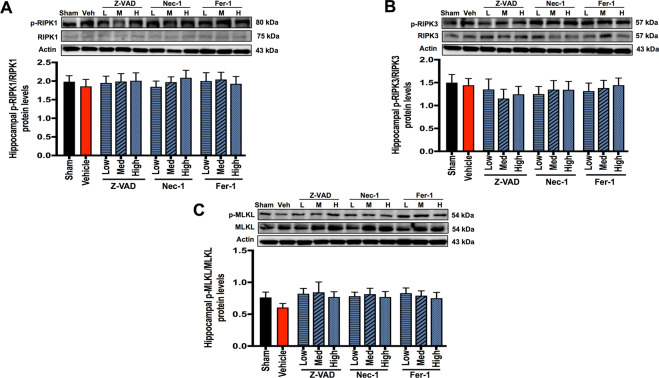
The effects of cell death inhibitors on hippocampal necroptosis signaling pathways. **A** p-RIPK1/RIPK1 protein expression; **B** p-RIPK3/RIPK3 protein expression; **C** p-MLKL/MLKL protein expression. Data are shown as mean ± SEM. *n* = 6/group. Veh: cardiac I/R rats treated with vehicle; L: cardiac I/R rats treated with low dose of cell death inhibitor; M: cardiac I/R rats treated with medium dose of cell death inhibitor; H: cardiac I/R rats treated with high dose of cell death inhibitor. I/R ischemia/reperfusion injury, RIPK receptor interacting protein kinases, MLKL mixed lineage kinase domain-like protein.

**Fig. 6:**
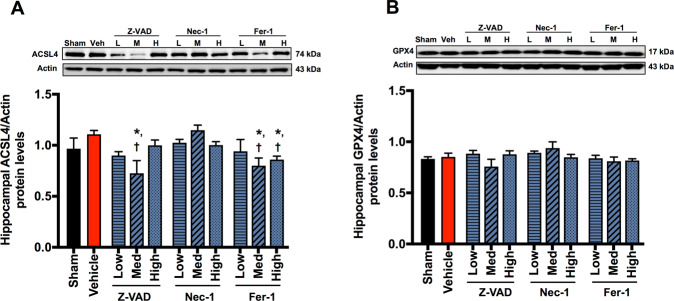
The effects of cell death inhibitors on hippocampal ferroptosis signaling pathways. **A** ASCL4 protein expression; **B** Gpx4 protein expression. Data are shown as mean ± SEM. *P < 0.05 vs. Sham, ^†^*P* < 0.05 vs. Cardiac I/R + Vehicle; *n* = 6/group. Veh: cardiac I/R rats treated with vehicle; L: cardiac I/R rats treated with low dose of cell death inhibitor; M: cardiac I/R rats treated with medium dose of cell death inhibitor; H: cardiac I/R rats treated with high dose of cell death inhibitor. I/R ischemia/reperfusion injury, ASCL4 acyl-CoA synthetase long-chain family member 4, Gpx4 glutathione peroxidase 4.

Cardiac I/R injury increased Bax/Bcl-2 and cleaved caspase-3/procaspase-3 protein levels, when compared with the sham group (Fig. 4C, D). The protein levels of p-RIPK1/RIPK1, p-RIPK3/RIPK3, p-MLKL/MLKL, ACSL4, and Gpx4 were not different between the sham and vehicle groups (Fig. 5A–C and Fig. 6A, B). These data suggested that only apoptosis occurred in the hippocampus following cardiac I/R injury.

Medium-to-high-dose Z-VAD and all doses of Nec-1 and Fer-1 markedly decreased Bax/Bcl-2 ratio, while low dose of Z-VAD was insufficient to reduce Bax/Bcl-2 ratio, when compared with the vehicle group (Fig. 4C). Cleaved caspase-3/procaspase-3 ratio is a marker of final apoptotic process. Our data showed that cleaved caspase-3/procaspase-3 ratio was inconsistent to Bax/Bcl-2 ratio. We found that medium-dose Z-VAD and all doses of Nec-1 significantly decreased cleaved caspase-3/procaspase-3 ratio, while the other treatment regimen (Fer-1) did not reduce cleaved caspase-3/procaspase-3 ratio (Fig. 4D). These data suggested that medium-dose Z-VAD and all doses of Nec-1 effectively reduced hippocampal apoptosis following cardiac I/R injury.

For necroptosis, none of these treatments altered p-RIPK1/RIPK1, p-RIPK3/RIPK3, or p-MLKL/MLKL in the hippocampus following cardiac I/R injury (Fig. 5A–C).

For ferroptosis, although ACSL4 was not increased in the vehicle group, medium-dose Z-VAD and medium-to-high-dose Fer-1 reduced ACSL4 protein levels, when compared with the sham and vehicle groups (Fig. 6A). Gpx4 protein levels were not different among groups (Fig. 6B).

## Discussion

The major findings of this study are as follows: (1) cardiac I/R injury causes brain damage as indicated by hippocampal dendritic spine loss, brain mitochondrial dysfunction, BBB breakdown, and increased hippocampal AD-related proteins, (2) cardiac I/R injury induced only hippocampal apoptosis, while necroptosis and ferroptosis did not occur, (3) cell death inhibitors reduced dendritic spine loss independently of cardioprotective effects, (4) brain mitochondrial dysfunction was partially attenuated by apoptosis and ferroptosis inhibitors, (5) AD-related protein levels and tau hyperphosphorylation were suppressed by all cell death inhibitors, (6) BBB proteins were preserved by necroptosis inhibitor, and (7) hippocampal apoptosis was reduced by both apoptosis and necroptosis inhibitors.

A reduction in cerebral blood flow (CBF) is an inciting event responsible for neuropathology in many diseases, including AMI [7]. Decreased brain oxygen and glucose supply, as a result of inadequate CBF, causes secondary brain injury, including neuroinflammation, oxidative stress, and cell death [19, 20]. Consistent with previous studies, we found that cardiac I/R injury led to brain mitochondrial dysfunction, BBB breakdown, amyloid beta aggregation, tau hyperphosphorylation, and subsequent dendritic spine loss [3, 4, 8, 9]. In addition, this is the first study to demonstrate that only apoptosis, but not necroptosis or ferroptosis, occurred in the hippocampus in rats with cardiac I/R injury.

Three different doses of apoptosis, necroptosis, and ferroptosis inhibitors were administered to the rats prior to cardiac ischemia. A pan-caspase inhibitor, Z-VAD, was selected as an apoptosis inhibitor. Although low-dose Z-VAD (1.65 mg/kg) reduced the myocardial infarct size, it was inadequate to exert neuroprotection in our study. On the contrary, medium- and high-dose Z-VAD attenuated dendritic spine loss possibly via reducing tau hyperphosphorylation. Although high-dose Z-VAD effectively reduced BACE1, it failed to reduce Aβ aggregation, possibly resulted from Z-VAD cytotoxicity. This finding might explain a lower efficacy in attenuating dendritic spine loss by high-dose Z-VAD, when compared with medium-dose Z-VAD, as several studies showed that the fluoromenthylketone compound of Z-VAD is cytotoxic when converted to fluoroacetate [21, 22]. Collectively, we have shown that an optimal dose of Z-VAD-FMK is required to exhibit the neuroprotection against cardiac I/R injury-induced brain damage. Our data suggested that medium-dose Z-VAD provided the best efficacy in reducing brain damage.

The medium-dose Z-VAD not only suppressed apoptosis but also reduced brain mitochondrial ROS levels and mitochondrial membrane depolarization. Aβ plaque, which is a product of BACE1-mediated APP cleavage, and tau tangle are hallmarks of AD pathology [23]. Our data found that medium-dose Z-VAD also reduced tau hyperphosphorylation, BACE1 levels, and Aβ aggregation, resulting in a higher efficacy in attenuating dendritic spine loss. Additionally, medium-dose Z-VAD reduced lipid peroxidation-induced ferroptosis as shown by decreased ACSL4 levels. Our results showed that zVAD.fmk effectively blocked caspase-3 cleavage when we administered at the dose of 3.3 mg/kg. However, lower dose (1.65 mg/kg) and higher dose (6.6 mg/kg) of zVAD.fmk did not block caspase-3 cleavage. We speculate that 1.65 mg/kg of zVAD.fmk was insufficient to inhibit caspase activity. For a high dose of zVAD.fmk, our data were consistent with a previous study; they demonstrated that zVAD.fmk treatment at 10 and 20 mg/kg did not reduce caspase-3 activity in pneumovirus-infected mice [24]. Therefore, we implied that the effective dose of zVAD.fmk is ranging between 2 and 6 mg/kg, when it was given peripherally. This speculation was supported by the data from previous studies, which suggested that treatment with zVAD.fmk at 2 and 5.6 mg/kg via intraperitoneal injection decreased caspase-3 activity, resulting in reduced brain edema and improved neurological function in rats with focal cerebral I/R injury and rats with subarachnoid hemorrhage [25, 26]. Apoptosis has been shown to occur following the Bax-mediated mitochondrial membrane permeabilization, subsequent cytochrome-c (cyt-c) release, and caspase-3 activation [27]. zVAD is a pan-caspase inhibitor which irreversibly binds to the catalytic site of caspases, leading to the suppression of caspase activity [22]. In addition, several studies demonstrated that zVAD could directly act upon Bax by inhibiting its activity or expression [28, 29]. Moreover, pretreatment with zVAD was shown to reduce the Bax expression in Chikungunya-infected HeLa cells [29]. These data supported our findings that zVAD could effectively reduce Bax as well as caspase-3 expression, leading to an attenuation of brain apoptosis in rats with cardiac I/R injury.

Nec-1 is a potent allosteric inhibitor of RIPK1 kinase activity [13]. It also inhibits indoleamine-2,3-dioxygenase (IDO), which is an enzyme involved in immunomodulatory function [10, 30]. Previous studies revealed that IDO is associated with neuroinflammation and neurodegeneration through a modulation of kynurenine metabolism quinolinic acid, contributing to cognitive impairment [31, 32]. In our study, all doses of Nec-1 failed to reduce myocardial infarction size. Moreover, it had no effect on RIPK1, RIPK3, or MLKL signaling in the brain despite its neuroprotective benefits following cardiac I/R injury. Thus, we speculated that the favorable effects of Nec-1 in our study are mediated through RIPK1-independent pathway.

Among three different doses of Nec-1, low-dose Nec-1 could provide neuroprotection. Low-dose Nec-1 effectively reduced apoptosis, tau hyperphosphorylation, BACE1 levels, and Aβ aggregation and preserved BBB tight junction protein, leading to attenuated dendritic spine loss. Although medium-to-high-dose Nec-1 showed similar effects to low dose, they could not suppress tau hyperphosphorylation. The association between Nec-1 and apoptosis is controversial [13]. Our findings are consistent with the previous study, showing that Nec-1 increased hippocampal Bcl-2 and decreased hippocampal cleaved caspase-3 in mice with traumatic brain injury [15]. We also found that Nec-1 did not suppress p-RIPK1, p-RIPK3, and p-MLKL in rat’s brain with cardiac I/R injury, since these three proteins were not upregulated in the brain following cardiac I/R injury, and Nec-1 did not disturb the physiological function of these proteins [17, 33]. Therefore, the protein expression of p-RIPK1/RIPK1, p-RIPK3/RIPK3, and p-MLKL/MLKL were not modified by Nec-1 treatment in our study. We speculated that the anti-apoptotic effects of Nec-1 are attributable to its neuroprotection in rats with cardiac I/R injury; however, the exact mechanism remains to be clarified.

Fer-1 attenuated the dendritic spine loss through a different mechanism from Z-VAD and Nec-1. Medium-to-high-dose Fer-1 inhibited ACSL4 protein levels, without affecting apoptotic protein levels. ACSL4, a contributing enzyme of ferroptosis, esterifies free polyunsaturated fatty acids (PUFAs) into PUFA-CoA, a key substrate for lipid peroxidation [34]. ACSL4 is abundantly expressed in the hippocampus compared to other areas of the brain [35, 36]. Previous study reported that ACSL4 plays an important role in dendritic spine architecture [37]. It affects axonal transport of synaptic vesicles and inhibits synaptic growth by altered lipids [38–40]. Redox-responsive transcription factor sp1, which is dysregulated in AD and cerebral ischemia, reportedly upregulates the expression of ACSL4 by directly binding to the ACSL4 regulatory region [41–44]. In our study, low-dose Fer-1 did not reduce dendritic spine loss although it suppressed AD-related proteins and tau hyperphosphorylation. On the contrary, dendritic spine was preserved by medium-to-high-dose Fer-1, in addition to decreased ACSL4 protein levels. This implicated that Fer-1 prevented brain damage through a suppression of ACSL4 and ferroptosis.

In addition, Bax and Bcl-2 are potentially central regulators of apoptosis, necroptosis, and ferroptosis as they serve as crosstalk between these cell death pathways [45–48]. Our data showed that Bax/Bcl-2 was remarkably decreased by all cell death inhibitors and might involve in neuroprotection following cardiac I/R injury.

The penetrability of each cell death inhibitor across the BBB should also be considered for their neuroprotection. Z-VAD and Fer-1 poorly cross the BBB in animal studies [49, 50]. In our study, although low-dose Fer-1 reduced tau hyperphosphorylation and Aβ aggregation, it could not reduce dendritic spine loss, and low-dose Z-VAD did not exert neuroprotection. On the other hand, Nec-1 was able to penetrate through BBB [51, 52], thus Nec-1 could reduce brain damage independent of cardiac effect.

In this study, the following antibodies that we used in the western blot analysis are polyclonal antibodies: APP, claudin5, TNF-α, occludin, p-RIPK1, MLKL, p-MLKL, Bcl-2, BACE1; monoclonal antibody should be considered to use in the future study to warrant our findings.

In conclusion, cell death inhibitors attenuated hippocampal dendritic spine loss caused by cardiac I/R injury through different mechanisms. Z-VAD promoted neuroprotection via ameliorating apoptosis and mitochondrial dysfunction. Nec-1 preserved BBB proteins and also reduced apoptosis, an effect that is beyond its RIPK1 inhibition, while Fer-1 suppressed ACSL4. All treatment effectively reduced AD-related proteins and tau hyperphosphorylation in the brain following cardiac I/R injury. Considering clinical significance in acute MI treatment, cell death inhibitor administration during ischemia or at the onset of reperfusion is more clinically relevant. Therefore, the effects of cell death inhibitors given during ischemia or at the onset of reperfusion should be further investigated.

## Materials and methods

### Experimental protocols

One-hundred and twenty-six male Wistar rats weighing between 400 and 500 g were acquired from Nomura Siam Company, Bangkok, Thailand. Rats were maintained in a temperature- and humidity-controlled room. Rats freely accessed the standard chow diet (CP082, Thailand) and water. The number of animals used in this study was chosen based on our previous studies [3, 4, 8].

Rats were anesthetized by intramuscular injection of Zoletil (50 mg/kg, Virbac, Thailand) in combination with Xylazine (0.15 mg/kg, LBS labs, Thailand). Then they were subjected to either the sham operation (*n* = 6) or the cardiac I/R operation (*n* = 120). Tracheostomy was done in all rats to facilitate ventilation with room air via positive pressure ventilator (CWE Inc., USA). The chest was open at the fourth intercostal space to expose the heart. The left anterior descending (LAD) coronary was identified, and the ligation site was marked with surgical suture, at approximately 2 mm distal to its origin. The rats in cardiac I/R groups were pretreated with either vehicle or cell death inhibitors in various doses 15 min prior to LAD ligation. LAD was ligated for 30 min, followed by 120 min of reperfusion. A successful LAD ligation was confirmed by an elevated ST segment on the electrocardiogram. Rats that did not develop an ST elevation were excluded from this study; however, all rats exhibited similar ST elevation and myocardial infarction after LAD ligation. Thus, randomization was not performed in this study. The group allocation was not blinded in this study. In the sham-operated group, the chest was opened for 165 min without LAD ligation [3, 4].

Rats in the cardiac I/R groups were divided into 10 subgroups as follows: (1) vehicle (10% dimethyl sulfoxide (DMSO) in normal saline solution), (2) low-dose Z-VAD (1.65 mg/kg), (3) medium-dose Z-VAD (3.3 mg/kg), (4) high-dose Z-VAD (6.6 mg/kg), (5) low-dose Nec-1 (1.65 mg/kg), (6) medium-dose Nec-1 (3.3 mg/kg), (7) high-dose Nec-1 (6.6 mg/kg), (8) low-dose Fer-1 (1 mg/kg), (9) medium-dose Fer-1 (2 mg/kg), and (10) high-dose Fer-1 (4 mg/kg). The inhibitor dissolved with 10% DMSO in normal saline solution were delivered to the rats via intravenous administration through a left femoral catheter. At the end of reperfusion, the rats were decapitated, and the brain and heart were removed.

**Fig. 7:**
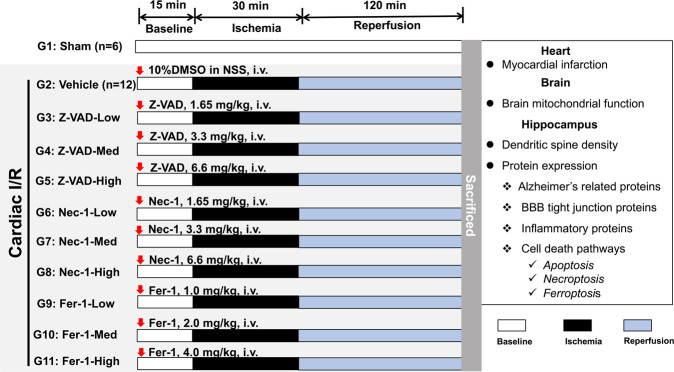
The experimental protocol of this study. One-hundred and twenty-six male Wistar rats were subjected to either sham operation (G1: sham, *n* = 6) or cardiac I/R operation (G2-G11, *n* = 12/group). Rats in cardiac I/R group were pretreated with either 10% DMSO in normal saline solution (G2: vehicle, *n* = 12) or cell death inhibitors, including an apoptosis inhibitor (Z-VAD), a necroptosis inhibitor (Nec-1), and a ferroptosis inhibitor (Fer-1). All inhibitors were subdivided into three different doses, including low, medium (Med), and high dose (*n* = 12/group). I/R ischemia/reperfusion injury, i.v. intravenous, DMSO dimethyl sulfoxide.

The heart was stained with Evans Blue-Triphenyl Tetrazolium Chloride dye to quantify the %infarct size/area at risk [3, 4]. The brain tissue was used to determine dendritic spine density, mitochondrial function, protein expression of BBB tight junction proteins, AD-related proteins, inflammation, and cell death-related proteins. The detailed experimental protocol is illustrated in Fig. 7.

### Brain mitochondrial function determination

The brain was homogenized immediately in an ice-cold buffer containing 225 mM Mannitol, 1 mM EGTA, 5 mM HEPES, 1 mg/ml bovine serum albumin (BSA), and 0.05% proteinase bacterial. The homogenate was centrifuged for 4 min at 2000 × *g*; the supernatant was then collected and centrifuged at 12,000 × *g* for 9 min. The pellet was resuspended with 0.02% digitonin in an ice-cold solution and centrifuged again at 12,000 × *g* for 11 min. All centrifugation protocols were performed at 4 °C. Mitochondrial protein concentration was determined by the bicinchoninic acid assay kit (Sigma, USA), and 0.4 mg/ml of mitochondrial protein was used for all mitochondrial function measurements [4, 9].

### Brain mitochondrial ROS levels

The isolated brain mitochondria were incubated with a dichlorofluorescin diacetate dye, which can be oxidized to dichlorofluorescein (DCF) in mitochondria. After incubation at room temperature for 20 min, the DCF fluorescence intensity was measured using a fluorescent microplate reader (the excitation wavelength was 485 nm and the emission wavelength was 530 nm). The DCF fluorescence intensity was used to represent mitochondrial ROS levels [4, 9].

### Brain mitochondrial membrane potential changes

The isolated brain mitochondria were incubated with 5,5′,6,6′-tetrachloro-1,1′,3,3′-tetraethylbenzimidazolcarbocyanine iodide (JC-1) dye. The monomer form of JC-1 (the excitation wavelength for green fluorescence was 485 nm and the emission wavelength was 530 nm) interacts with anions in the mitochondrial matrix, becoming an aggregated form, which is detected as a red fluorescence (the excitation wavelength for red fluorescence was 485 nm and the emission wavelength was 590 nm). Red/Green ratio of JC-1 was used as an indicator of mitochondrial membrane potential changes. A decrease in this ratio implicates mitochondrial membrane depolarization [4, 9].

### Brain mitochondrial swelling

The absorbance value of the mitochondrial suspension at 540 nm was measured via a microplate reader. The decrease in mitochondrial absorbance indicated brain mitochondrial swelling [4, 9].

### Protein expression analysis

Hippocampal tissues were used to determine the protein expression of AD-related proteins, BBB-related proteins, inflammation, and cell death pathways. The hippocampal tissues were lysed with lysis buffer and the protein concentration was measured using a Bio-Rad protein assay kit (Bio-Rad Laboratories, USA). Protein was separated by 10% sodium dodecyl sulfate-polyacrylamide gel electrophoresis and subsequently transferred to nitrocellulose membranes. The membranes were blocked for 1 h in either 5% skimmed milk or 5% BSA in Tris-buffer saline (pH 7.4) containing 0.1% Tween 20. The membranes were subsequently incubated with the primary antibodies (overnight, 4 °C) against APP (Cat.# 2452, 1:1000 dilution, Cell Signaling Technology [CST]), Aβ (Cat.# sc-28365, 1:1000 dilution, Santa Cruz Biotechnology [SCBT]), Tau and p-Tau (Cat.# 4019, Cat.# 12885, 1:1000 dilution, CST), NF-κB and p-NF-κB (Cat.# 8242, Cat.# 3033, 1:1000 dilution, CST), TNF-α (Cat.# ab9635, 1:1000 dilution, Abcam), claudin-5 (Cat.# ab15106, 1:1000 dilution, Abcam), occludin (Cat.# sc-5562, 1:1000 dilution, SCBT), RIPK1 and p-RIPK1 (Cat.# 3493, Cat.# 31122, 1:1000 dilution, CST), RIPK3 (Cat.# 15828, 1:1000 dilution, CST), p-RIPK3 (Cat.# ab195117, 1:1000 dilution, Abcam), MLKL and p-MLKL (Cat.# PA5-43960, Cat.# PA5-105678, 1:1000 dilution, Invitrogen), Bax and Bcl-2 (Cat.# ab182733, Cat.# ab196495, 1:1000 dilution, Abcam), caspase-3 and cleaved caspase-3 (Cat.# 14220, 1:1000 dilution, CST), Gpx4 (Cat.# ab125066, 1:1000 dilution, Abcam), ACSL4 (Cat.# sc-365230, 1:1000 dilution, SCBT), BACE1 (Cat.# ab2077, 1:1000 dilution, Abcam), SOD2 (Cat.# 13194, 1:1000 dilution, CST), and β-Actin (Cat.# sc-47778, 1:1000 dilution, SCBT). Then they were incubated with horseradish peroxidase–conjugated secondary antibodies for 1 h at room temperature. The membranes were visualized by Clarity TM Western ECL Blotting Substrate (Bio-Rad Laboratories, USA). The western blot images were taken with the ChemiDoc Touching system (Bio-Rad, USA), and they were analyzed by the Image J analysis software (NIH image) [3].

### Dendritic spine density determination

After decapitation, the whole brain was removed and rinsed with cold phosphate-buffered saline (PBS). Then the brain was sliced using a vibratome (Vibratome Company, USA) at 400 μm thickness. All brain sections were fixed with 4% paraformaldehyde for 1 h and were washed with PBS. Then brain sections were incubated with 1,1’-dioctadecyl-3,3,3’,3’-tetramethylindocarbocyanine perchlorate dye (DiI dye; Invitrogen) for 1 week at room temperature. A confocal microscopy (Olympus fluoview FV3000) was used to acquire neuronal images in the CA1 hippocampus area. Dendritic spine density was analyzed by the Imaris software 7.0 (Bitplane, Oxford instrument company, Switzerland).

### Statistical analysis

Data for each experiment were expressed as mean ± SEM and processed using GraphPad Prism 8 (GraphPad Software, Inc., USA). A one-way analysis of variance followed by post hoc Fisher’s least significant difference test was used to analyze the significance of difference between groups. *p* value < 0.05 was considered statistically significant.

## Data Availability

The data that were analyzed during the current study are available from the corresponding author on reasonable request.
